# Delineating Potential Transcriptomic Association with Organochlorine Pesticides in the Etiology of Epithelial Ovarian Cancer

**DOI:** 10.2174/1874091X01812010016

**Published:** 2018-02-28

**Authors:** Harendra K. Shah, Muzaffer A. Bhat, Tusha Sharma, Basu D. Banerjee, Kiran Guleria

**Affiliations:** 1 Environmental Biochemistry and Molecular Biology Laboratory, Department of Biochemistry, University College of Medical Sciences & G.T.B. Hospital (University of Delhi), Dilshad Garden, Delhi 110095, India; 2 Department of Obstetrics and Gynecology, University College of Medical Sciences & G.T.B. Hospital (University of Delhi), Dilshad Garden, Delhi 110095, India.; ^3^Department of Physiology, All India Institute of Medical Sciences, New Delhi 110029, India

**Keywords:** Epithelial ovarian cancer (EOC), Gene expression, Gas chromatography (GC), Microarray, Organochlorine pesticides (OCPs), Heptachlor epoxide B (HTEB)

## Abstract

**Background:**

Recent studies have shown that there is an increased risk of Epithelial Ovarian Cancer (EOC) with Organochlorine Pesticides (OCPs). However, the alteration in the gene expression profile has not been explored so far. The goal of the present study is to understand the probable molecular mechanism of OCPs toxicity towards discovery of dysregulation of signaling pathway associated with differential gene expression and candidate transcriptomic set of markers in the pathophysiology of EOC in OCPs exposed population.

**Methods:**

The OCP levels were estimated by gas chromatography and whole genome differential expression study was carried out using expression microarray and candidate genes were validated using Real time RT-PCR.

**Results:**

Significant level of OCP residues such as β-hexachlorocyclohexane (β-HCH), Heptachlor, Heptachlor epoxide B (HTEB), dichlorodiphenyldichloroethylene (p’p’-DDE) and endosulfan-I was found between healthy and EOC patients. The transcriptome profile of several genes revealed regulation of various important cellular processes such as metabolism, inflammation, cytoskeleton dysregulation of TGF and WNT pathway in EOC cases with high OCPs.

**Conclusion:**

This study provides the first evidence showing that differentially expressed genes and dysregulation of signaling pathways might be associated with significant level of OCPs exposure in ovary tissue of epithelial ovarian cancer patients. Moreover, significant correlation of these genes with OCPs revealed that OCPs exposure played vital role in dysregulation of related pathways in the etiology of EOC

## 1 INTRODUCTION

Ovarian cancer is the fifth most leading cause of cancer death worldwide with highest mortality rate than any other cancer of the female reproductive system [1]. In India, ovarian cancer has been found to be the third leading cause of cancer among women [2]. 80-90% of the ovarian cancer cases comprises of the Epithelial Ovarian Cancer (EOC) [3]. The etiopathology of EOC is multifactorial, including environmental, endocrinological, dietary factors and genetic factors [4-9].

Many risk factors associated with Epithelial Ovarian Cancer (EOC) pathology are still not completely known. There is evidence to suggest that Organochlorine Pesticides (OCPs) constitute one of the major environmental factors for EOCs [10]. OCPs have been widely used in last six decades or more contributing to the state of environmental pollution today [11]. In India, OCPs are mostly used in agricultural practice and public health program as insecticides, herbicides and fungicides [12, 13]. More than 98% of sprayed insecticides and 95% herbicides reach air, water, bottom sediments, food and non-target living system other than targeted species [14].

OCPs demonstrate endocrine disrupting properties both *in vivo* and *in vitro* studies [15, 16]. OCPs are highly lipophilic and resistant to biotransformation in nature, they tend to accumulate in fatty tissue, adipose tissue where they may alter endocrine function by mimicking hormone, blocking the effects of normal endogenous hormone or may modify the synthesis, metabolism and transport of hormones [17]. Their long half lives in human body up to several decades make them susceptible chronic pathogenesis such as prostate cancer, urinary bladder cancer, breast cancer and epithelial ovarian cancer [18-21]

Despite indirect evidence that OCPs may act to result in EOCs, there is no robust report to suggest how these chemicals may influence genomic expression in the pathogenesis of EOCs. Some recent studies indicate that these organochlorine pesticides may alter the expression of several transcripts across the genome, thus resulting in many etiopathologies such as cancer [22-24]. Since there seems to be a rising incidence of ovarian cancer cases in North India [25, 26] and also the rampant use of organochlorine pesticides in this region [27-29], it becomes imperative to understand transcriptomic expression changes in ovarian cancer subjects in conjunction with their tissue OCP levels. Thus, the present study aims to explore the transcriptomic expression profile of the EOC tissue and determine the association, if any, with their respective tissue OCP levels.

We report here for the first time that differential gene expression along with various cellular- molecular pathways dysregulated in high OCPs exposed EOC. Based on present transcriptomics data, we have hypothesized that dysfunctional xenobiotic metabolizing enzymes (XME)-inflammatory-cytoskeleton-TGF-WNT-RhoGTPase behaviors may be associated with EOC in high OCPs exposure.

## 2 METHODS

### 2.1 Subjects and Tissue Samples

In the present study, Nineteen (19) subjects with confirmed EOC were recruited and thirty (30) age matched controls were recruited with similar life style-(Supplementary Table **1**). Subjects of EOC were included on the basis of clinical examination, imaging and confirmed by cytological or histopathological examination. Women with other types of ovarian cancers (non-epithelial in origin) or other benign reproductive disorders, diabetes, etc. were excluded from the study. Premenopausal women admitted for uterovaginal prolapse surgery or Pan Hysterectomy was taken as control. Ovaries of the control subjects were confirmed histologically to be non-cancerous. Women who were using talcum powder, tobacco and environmental exposure other than pesticides were excluded from the study. The study was approved by Institutional Ethical Committee for Human Research, University College of Medical Sciences & GTB Hospital (reference no UCMS/IEC-HR/2013). All the patients had voluntarily agreed to donate their samples after understanding the purpose of the proposed study. Written informed consent was obtained from each subject prior to their inclusion in the study as per ethics norms. The plan of the study is illustrated in Fig. (**1**).

#### 2.1.1 Sample Collection

Approximately 1.5gm of EOC samples and control ovarian tissue specimens were collected in sterile ice cold phosphate buffered saline (PH 7.4) immediately after surgery. 1gm of the obtained tissue was processed for pesticide estimation and rest was processed for RNA extraction. RNA was stored at -80°C for microarray and Real time RT-PCR.

### 2.2 Extraction of Organochlorine Pesticides

All the chemicals used in the study were of high-purity grade. The Extraction of OCP residues was done as shown in Supplementary Fig. (**1**). Briefly, ovary tissue was homogenized and mixed with Hexane and Acetone in the ratio of 5:2. Then it is spin in the shaker for 4hrs and supernant was collected. The same procedure carried out twice. Cleanup was done by the USEPA method using Florisil (Sigma) by column chromatography. Thus obtained pure extract was evaporated and finally mixed with 1ml of Hexane and then quantified. Quantification of OCP residue levels has been done using high resolution gas chromatography (Clarus^®^500, Perkin Elmer, USA) equipped with ^63^Ni electron capture detector and an Elite GC DB-5 capture column of 60mm x 0.25mm I.D. capillary column (J&W scientific, folsom, CA, USA) was used throughout the analyses. The standard operating procedures of GC and protocols were followed as earlier study [30]. For quality control, five samples in triplicate were spiked with 5, and 25 ng/ml of a mixed standard of OCPs. The average recovery of fortified samples was more than 95%. Furthermore, a quality sample check was always run with each set of samples for pesticide analysis to maintain accuracy.

The OCPs residues that could be detected in the ovarian tissue samples were: α, β-hexachlorocyclohexane (α, β-HCH); 1,1,1- trichloro-2,- (p- chlorophenyl)-2-(o-chlorophenyl)ethane (o’p’ DDT); 1,1-dichloro-2,2-bis(p-chlorophenyl) ethylene (p’p’ DDE); Heptachlor epoxide B (HTEB) and Heptachlor epoxide A (HTEA), heptachlor, dieldrin, endosulfanI, endosulfan II. Other OCPs that were detected in very few samples, have not been considered here.

### 2.3 Extraction of Total RNA

Total RNA was extracted from epithelial ovarian tissue samples using Trizol reagent (Invitrogen, Carlsbad, CA, USA) as previously described elsewhere [31]. The yield and quality of the extracted RNA was estimated using Nanodrop (Thermo scientific, Waltham, MA, USA). RNA integrity as assessed by the Agilent 2100 Bio Analyzer using the RNA 6000 Nano Lab Chip kit and Agilent 2100 Expert Soft-ware (Agilent Technologies, Santa Clara, CA, USA). RNA used for microarray and Real time RT-PCR.

### 2.4 Microarray

To understand the probable molecular mechanism of OCPs toxicity and to find out differential expression pattern of genes, eight diagnosed EOC (mean age 53.9) and seven control (mean age 48.57) ovarian tissues were selected for Whole Human Genome Microarray experiment (Supplementary Table **2**). The level of OCPs was equally distributed within samples of EOC and within control samples as shown in supplementary Fig. (**2**). So randomly Eight EOC and seven control ovary tissue samples were chosen. The samples with RIN scores >8 were subjected to whole genome expression microarray using the Agilent Whole Human Genome 60-mer oligonucleotides probes in 4X 44 K Microarray format by one color expression microarray based gene expression analysis [31]. Briefly, total RNA was used for the synthesis of double stranded cDNA by using High capacity cDNA Reverse Transcription using one color quick amplification kit (Agilent Technologies, Santa Clara, CA, USA). Cy3 labeled cRNA was synthesized using one color quick amplification kit. Cy3 labeled cRNA was purified by Qaigen’s RNeasy mini spin columns (Qiagen Inc., Valencia, CA, USA). cRNA was fragmented and hybridized to the microarray for 17h at 65°C. The microarray 4X44K slides washed, and subsequently scanned with Agilent’s G2505B microarray scanner system. The raw image was then imported into feature extraction software v 10.7.3.1 (Agilent Technologies) and the raw files were imported into GeneSpring 13.1.1 software (Agilent Technologies, Santa Clara, CA, USA).

### 2.5 Gene Expression Data Analysis

The statistical analysis was performed using the GeneSpring 13.1.1 software. The Principal Component Analysis (PCA) was performed to assess the distribution of samples on the basis of gene expression patterns with their underlying variability. The unsupervised hierarchical clustering algorithm using the Pearson correlation was then used to group the probe sets based on their expression pattern. Differential gene expression was compared using Welch t test with Benjamini-Hochberg corrections for false discovery rate

### 2.6 Post-hoc Analysis

Networks and enrichment analysis were done using differentially expressed gene lists obtained from the above analyses with the help of the GeneSpring13.1.1 software and MetaCore platform (GeneGo, St. Joseph, MI, USA). To enrich our pathway analysis, we selected gene sets that represented a cut-off threshold (two or more fold chain with *p*< 0.05). We used the “Meta Core Analysis” function to interpret the data in the context of biological processes, pathways and biological networks. Further dysregulation of gene expression and related changes in bio-functions under the subcategories of Signaling & Metabolic functions, Cellular & Molecular functions were identified. Canonical pathway analysis identified specific functional genes significantly present within the networks.

### 2.7 Validation of the Genes by Real time PCR

The differential expression of 13 genes (TGF β RII, NKIRAS1, TXNRD2, CKLF, TNFRSF11A, PTPRC, IL37, CCL23, CCR2, G3BP2, HNMT, IGFBP7, and UBE2V1) were validated in 19 EOC and 30 control ovary tissue samples by Real time RT-PCR. These genes were chosen on the basis of known, probable or putative involvement in cancer (Supplementary Table **3**). The primers were designed by Beacon Designer. The sets of forward and reverse primers used for these genes are given in Supplementary Table **4**.

The reverse transcription reaction was performed according to the protocol of the Verso cDNA synthesis kit (Thermofischer Scientific) as per The MIQE Guidelines. The raw florescence data were analyzed using Bio RAD Manager Software. GAPDH (Glyceraldehyde-3-phosphate dehydrogenase) and β actin were selected as endogenous controls. The geometric mean of these two endogenous controls was used for normalizing the mRNA levels for the gene of interest. The geometric mean of C_T_ of endogenous controls was subtracted from the C_T_ of the respective gene_,_ followed by subtraction of the median control group ∆C_T_ value, giving the ∆∆C_T_ [32].

### 2.8 Data Analysis

The statistical analysis was carried out by using SPSS ver 21.0. OCPs Data are expressed in the form of mean ± standard deviation (SD). Unpaired student's t-test and chi square test was applied to compare socio-demographic characteristics. As pesticides are not normally distributed hence non parametric Mann-Whitney test was used to compare pesticide levels in cases and controls. Correlation analysis between the differential gene expression and OCPs among healthy and EOC patients was done by Pearson correlation. Differential gene expression was compared using Welch unpaired t-test. The *p* value of <0.05 was considered as statistically significant.

## 3 RESULTS

Various parameters like, age, body mass index (BMI), menstrual history which are supposed to be risk/protective factor for EOC were taken into consideration to assess their association with OCPs exposure. BMI values were higher in EOC patients than in healthy women (*p*< 0.006) which show pesticides content in individual is proportional to their fat content as pesticides accumulate in the fatty tissue [17]. However, we did not find any significant difference in age, menopause and other socio-demographical features between EOC patients and control (Supplementary Table **1**).

### 3.1 Comparison of OCPs Between Control and EOC Patients

We found significant level of β-HCH (*p*<0.001), Heptachlor (*p*<0.001), HTEB (*p*<0.001), p,p’ DDE (p<0.001) and endosulfan I (*p*<0.001) in ovarian tissue of EOC patients as compare to control (Table **1**).

### 3.2 Global View of Changes in Gene Expression between EOC and Control Group

**Fig. (3):**
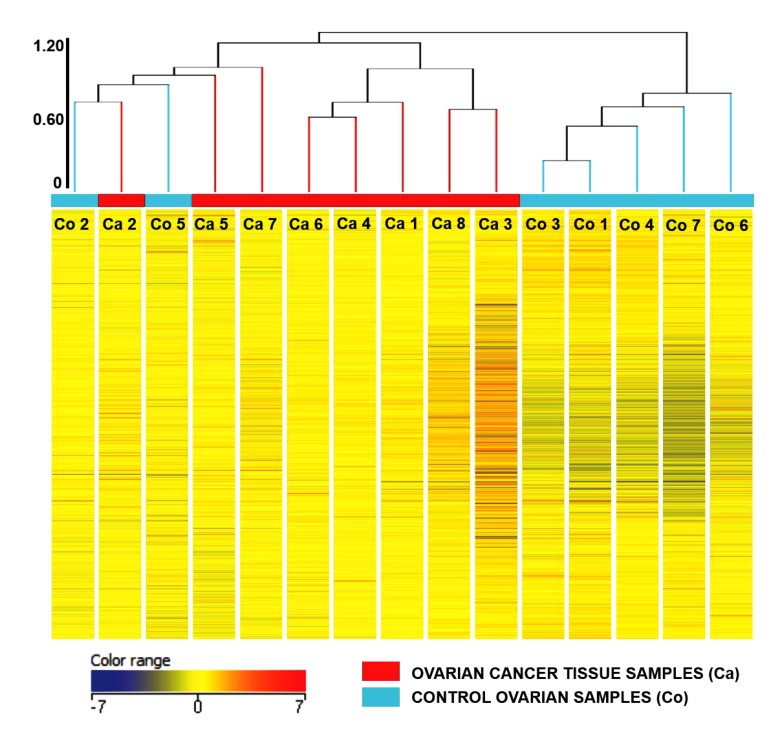
Two-way representation of unsupervised hierarchical cluster analysis (HCA) of the expression levels (in logarithmic scale) of all the target probes/genes (X-axis) in each sample (each row), EOC labeled as cancer in red and control labeled as control in sea green color from all subjects (n = 15) and their clustering based on expressional distance (Pearson correlation coefficient) between samples in dendrogram formation (Y-axis). Each horizontal line represents a single probe, and each column represents a single sample. Relative expression of each probe is color-coded: high (red) and low (blue), as indicated in the color legend.

Principal Component Analysis revealed a distinct cluster of all 8 EOC samples separate from the control samples. Principal component analysis determined differences in overall gene expression between the two groups of the microarray data set. Fig. (**2**) indicates the relationships between individual samples of these two groups categorized into 2 distinct areas showing distribution of 8 EOC samples represented by red dot in one circle and 4 control samples represented in sky blue and rest of the samples distributed separately. Hierarchic clustering depicted clustering nature of individual samples in these two groups (Fig. **3**)

### 3.3 Differential Gene Expression between EOC and Control Group

Supplementary Table **5** gives the list of the up regulated and down regulated genes along with their respective fold changes based on pairwise analysis of overall gene expression profiles between these two groups. Collectively, a total of 159 genes were differentially regulated in which 146 genes were up regulated and 13 genes were down regulated under FC >2.0 with *p* <0.01

### 3.4 Post Hoc Analysis

Gene set enrichment analysis was performed using GeneGO MetaCore (Thompson Reuters) and affected pathway maps, process networks and gene ontology were determined from the input differentially regulated genes between high OCPs exposed EOC and control ovary tissue samples.

#### 3.4.1 Pathways

Affected pathways included cytoskeletal remodeling-TGF/WNT/ Rho GTPases, Development-BMP7 in brown adipocyte differentiation, cell cycle spindle assembly and chromosome separation, Cell adhesion_Integrin-mediated cell adhesion and migration (Table **2**). Cytoskeleton remodeling occurred through more than one pathway such as TGF β, WNT and also by Rho GTPase suggesting the importance of this process in cancer.

#### 3.4.2 Gene Ontology

Over represented ontologies among genes revealed cellular metabolic process, organic substance metabolic process, primary metabolic process, cellular component organization (Table **3**).

#### 3.4.3 Gene Networks

Functional analysis of differentially regulated genes identified top score biological networks with the shortest path algorithm and relative saturation of networks with canonical pathways revealing 3 top score gene networks. The top process related to network 1 was MyD88 dependent toll like receptor signaling pathway with highest score of 239.61 which includes TRAF6, MyD88, I-kB, MAP3KI4, MAP2K3 as differentially regulated genes, the top process related to network 2 was wnt signaling with g score of 136.59 having VEGF-A, TCF7/LCF, P21, Dsh, Tcf4, Axin, DKK3, DKK1,DVL1, WNT as differentially regulated genes and those related to network 3 are TGF- β receptor signaling with g score of 61.76 having ESR2, NFYA, CDC25A, SMAD3, TGFβ R II (Table **4**).

### 3.5 Quantification of Candidate Gene Transcripts

Quantification of 13 candidate gene transcripts by real time RT-PCR followed by correlation coefficient revealed higher degree of concordance (r=0.797) between Microarray and Real time RT-PCR data in EOC compared with control ovary tissue samples (Supplementary Fig. **3**).

### 3.6 Organochlorine Pesticides and their Association with Gene Involved in EOC

Possible toxicological effects of OCPs on differential expressed genes TGF β RII, NKIRAS1, TXNRD2, CKLF, TNFRSF11A, PTPRC, IL37, CCL23, CCR2, G3BP2, HNMT, IGFBP7 and UBE2V1 was evaluated. A highly significant correlation was observed between levels of β HCH, heptachlor, HTEB, ppDDE and endosulfan1 with NKIRAS1, TXNRD2, CKLF, TNFRSF11A, PTPRC, IL37, CCL23, CCR2, G3BP2, HNMT, IGFBP7, and UBE2V1 genes. In addition ccr2 showed significant correlation with HTEA. However IGFBP7 did not show correction with HTEB as shown in Supplementary Table **6**.

## 4 DISCUSSION

The North India especially Delhi region is one of the most OCPs contaminated areas due to negligence in disposal of OCPs from farm and chemical manufacturing units to Yamuna River water and subsequently drinking water and vegetables. It has been already noted that OCPs were found significantly concentrated in river, food and drinking water in North India [27-29]. The above cited studies were already convinced by one of our earlier published work in which it has been showed the use of ground water as drinking water source was significantly associated with the etiology of EOC [9]. The present study is in agreement and reflect the possible route of pesticide exposure to the human population but the significant association of ground water as drinking water source could not have been achieved which may be due to smaller sample size.

On the basis of OCPs level, it has been observed that out of 19 cases of EOC majority of cases have high OCPs levels (> 60% tile of total OCPs) and number of cases with low OCP levels are very few (n=1) which may be due to incidental (Supplementary Fig. **2**). So, on the basis of non-uniform distribution of samples, no significant difference was observed between these two groups. However, one of the limitations of the study is the sample size particularly if we consider different grade and pesticide level. Hence further study are required to take up the larger sample size which can be further divided on the basis of different levels of pesticide with grading of EOC. Moreover, these limitations are not interfering to explain our present hypothesis that OCPs are one of the potential risk factor for EOC and may responsible for the dysregulation of genes.

The results of the present study have shown significantly higher levels of β-HCH, heptachlor, HTEB, p'p'-DDE and endosulfan-I in ovary tissue sample of EOC as compare to control which is in consistent with the earlier finding [9]. The presence of significant high level of these OCPs in the ovary tissue may act as endocrine disruptive pesticides (EDP) which can lead to hormonal imbalance and initiate or promote the mitogenic pathways. As a result, the response cascade of natural hormones can either inhibited or excessively enhanced at the wrong time, may play critical role in the cellular & molecular changes [33].

We are reporting for the first time, the differential expression of genes such as TGF β RII, NKIRAS1, TXNRD2, CKLF, TNFRSF11A, PTPRC, IL37, CCL23, CCR2, G3BP2, IGFBP7, and UBE2V1 involve in the etiology of EOC (Supplementary Table **5**). CCR2 gene was found up-regulated with highest fold change. Although, CCR2 has been implicated in range of inflammatory disease including rheumatoid arthritis, tumors, we are reporting for the first time that high expression of CCR2 may be due to high OCPs exposure in EOC sample. Insulin-like growth factor-binding protein 7 (IGFBP7) function as a tumor suppressor and involved in several cellular processes, including proliferation, senescence and apoptosis. Loss of IGFBP7 expression is a critical step in the development of human tumors [34]. IGFBP7 gene was down regulated, which are in consistent with earlier study [35]. However, dysregulation of these genes due to high OCPs exposure in the risk factor of EOC was not found in the previous literatures. This is the first study indicating OCPs have major impacts in mediating toxicities with the dysregulated genes involved cellular and molecular functions in developing EOC. Moreover, significant correlation between OCPs and the differential expressed genes supports and strengthen the hypothesis that predisposition of OCP in human body may altered the genetic make of an individual which may ultimately antedate the pathogenesis of EOC.

Several pathways which are characteristic of cancer were identified in this study such as cytoskeleton remodeling pathway which acts through the subsets of TGF/WNT/Rho GTPase signaling cascade between normal ovarian surface epithelium and epithelial ovarian cancer (Table **2**) [36]. Endosulfan disrupts WNT/β catenin signaling pathway [37] and activates Rho-GTPase proteins. This event linked to cancer cell migration/invasion during metastasis via the control of cytoskeletal remodeling [38]. DDT and its metabolite appear to promote multiple cancer-related processes including sustained proliferation through different pathways, such as TGF β, Wnt/β-catenin [39, 40].

Heptachlor has been found to trigger significant proliferation and tumor genesis in rat hepatocytes and inhibit apoptosis by activating MAPKK signaling pathways [41]. This study showed MAPK- MyD88- dependent toll like receptor signaling pathway with significantly altered genes such as TRAF6, MYD88 (Table **4**). Activation TRAF6 through MyD88, stimulate the phosphorylation of p38 via MAPK pathways leading to subsequent expression of inflammatory genes mediates various inflammatory responses [42].

Cytochrome P 450 (CYP 450) involved in metabolic activation and/or detoxification of various pro-carcinogens like pesticides. This study showed dysregulation of cellular metabolic process genes; CYP3A5, CYP39A1, cytochrome C, GSTM1, GSTA1, CYP2F1, CYP2A7, and CYP19 (Table **3**) which is in consistent with Downie *et al.* demonstrated higher levels of various CYP450 genes [43]

The effect of OCPs observed in the etiology of EOC, requires further validation by in vitro approach, exposing primary epithelial ovarian cells with OCPs to study the effect of OCPs exposure on gene expression by expressional microarray. This study is currently underway in our laboratory.

## CONCLUSION

These results indicate that β-HCH, Heptachlor, Endosulfan-I and DDT metabolites found in the ovary tissue could alter the genes involved in several pathways such as cytoskeleton remodeling/TGF-WNT/Rho GTPase and MAPK as well as cellular metabolic process. All these events account, at least in part, for the carcinogenic potential of β-HCH, Heptachlor β Endosulfan in ovary. Furthermore, the high risk population can be identified by estimating the exposure levels of OCPs and accordingly it might provide safety measures to be taken up.

## Figures and Tables

**Fig. (1):**
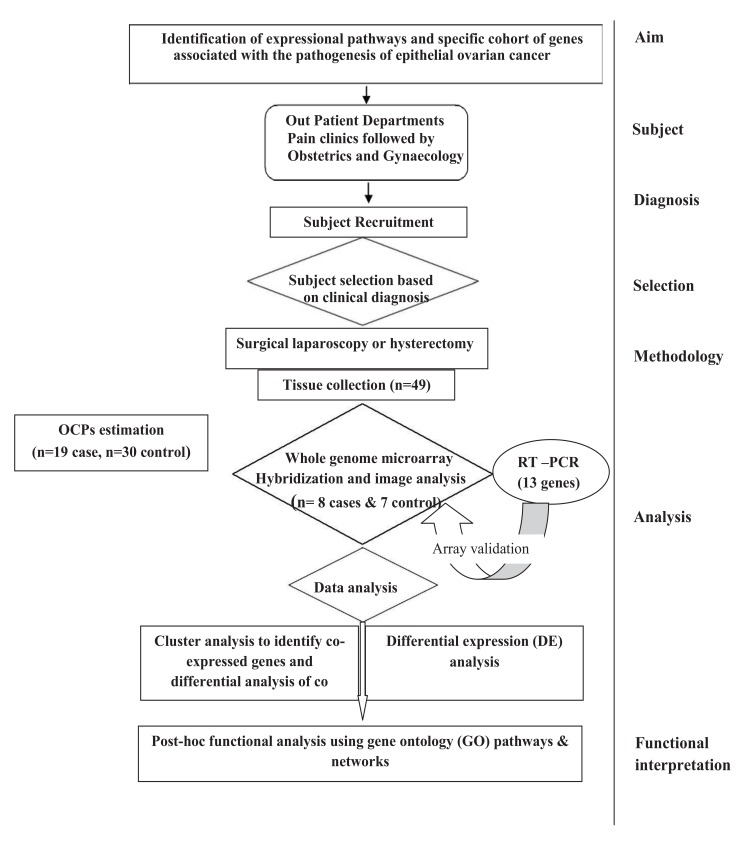
Flow diagram of the experimental design showing work plan of the present study.

**Fig. (2):**
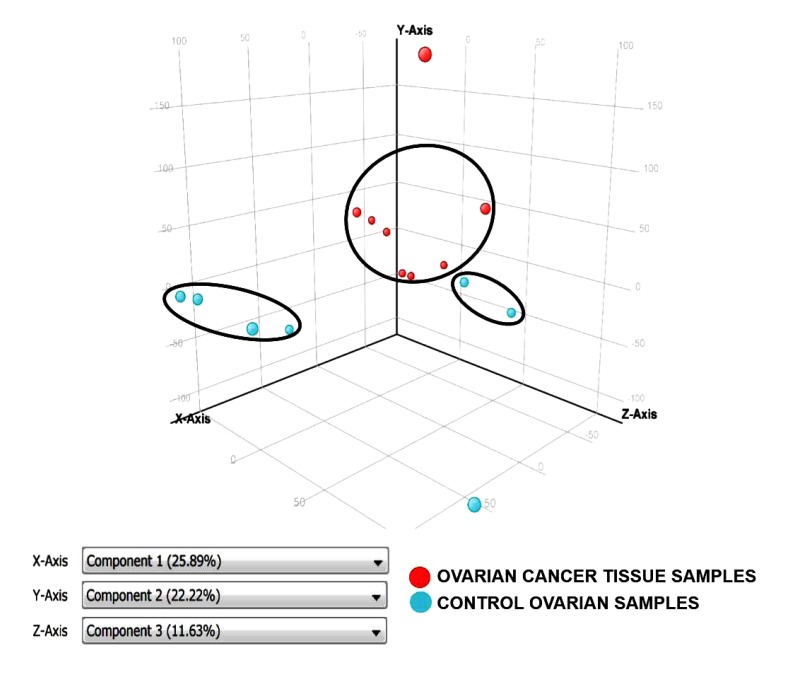
PCA of 8 EOC and 7 control ovary tissue samples. Unsupervised PCA was applied to the samples based on variance in gene expressions. The relative positions of individual EOC (shown in red color) and control (sky blue color) samples.

**Table 1:** Comparison of Organochlorine Pesticides (OCPs) level between epithelial ovary cancer tissue sample and control.

**OCPs** (ng/ml)	**Control** (n=30) Mean ±SD	**Case** (n=19) Mean ±SD	***P* Value**
α-HCH	2.22±1.04	2.66±1.24	0.191
β-HCH	3.33±1.03	6.21±1.31	0.001*
Heptachlor	4.53±1.58	11.19±2.12	0.001*
HTEB	2.72±1.22	4.95±1.15	0.001*
HTEA	1.42±1.03	1.63±0.96	0.479
o,p’-DDE	2.41±1.50	2.05±1.07	0.321
p,p’-DDE	1.77±1.28	4.75±1.42	0.001*
Endosulfan-I	2.15±1.00	4.09±1.60	0.001*
Endosulfan-II	2.14±1.13	2.72±1.12	0.087
Dieldrin	2.26±0.89	2.65±0.88	0.135

Asteric indicates level of significance at p<0.05. HCH: hexachlorocyclohexane ; o’p’ DDT: 1,1,1- trichloro-2,- (o- chlorophenyl)-2-(p-chlorophenyl)ethane; p’p’ DDE: 1,1-Dichloro-2,2-bis(p-chlorophenyl) ethylene; HTEB: Heptachlor epoxide B and HTEA: Heptachlor epoxide A.

**Table 2:** Over represented pathways^1^ among genes dysregulated in the EOC.

**Metacore Pathway Maps**	**Genes in Network**	***p* value**
Cytoskeleton remodeling	MELC, MRLC, MyHC, DOCK1, SMAD3, Rac1, VEGF-A, Tcf(Lef), eIF4G2, SOS, PI3K cat class IA, LIMK2, TGFβR II, Collagen IV, Nucleolin, EK3(MAP2K3), p38, MAPK, PTEN, Actin cytoskeletal, MLCP (cat)	3.699E-07
Cytoskeleton remodeling by TGF and WNT	MELC, MRLC, DOCK1, SMAD3, Axin, Rac1, VEGF-A, TCF7L2 (TCF4), Tcf(Lef), Dsh, SOS, PI3K cat class IA, LIMK2, TGFβR II, Collagen IV, WNT, Nucleolin, MMP-7, MEK3(MAP2K3), p38 MAPK, Actin cytoskeletal, Actin	4.728E-07
BMP 7 in brown adipocyte differentiation	CIDE-A, COL5A1, UCP1, TrkC, PPARGC1 (PGC1-alpha), EDNRA, Cytochrome c, DLK, BMPR1A, PPAR-gamma, WNT10A, ELOVL3, SERPINA3 (ACT)	1.219E-06
Cytoskeleton remodeling by Actin cytoskeletal	MELC, Myosin II, MRLC, MyHC, Rac1, LIMK2, Actin, MLCP (cat), actin cytoskeleton by Rho GTPases CDO1, Rac1, PP1-cat, SOS, PI3K cat class IA,	1.239E-05
Transcription by CREB Pathway	Galpha(s)-specific amine GPCRs, LDHA, IGF-1 receptor, G-protein alpha-s, Calmodulin, MEK3, p38 MAPK, Shc	2.056E-05
AVP in regulation of Aquaporin 2 & renal water reabsorption	Myosin II, cAMP-GEFI, MRLC, Non-muscle myosin IIA, MyHC, VAMP3, G-protein alpha-s, Calmodulin, Adenylate cyclase type VI, Adenylate cyclase type III, Actin cytoskeletal, ACTB, V2 receptor	2.604E-05
Regulation of GSK3 β in bipolar disorder	TrkC, Axin, PP2A regulatory, PP1-cat, Dsh, DVL-1, SOS, WNT, TrkA, IL-1 beta, Shc, Neutral sphingomyelinase	5.128E-05
Cell cycle	Tubulin alpha, Tubulin (in microtubules), Aurora-A, KNSL1, CDC20, Dynein 1, cytoplasmic, Aurora-B, ZW10, RCC1, DCTN2	5.386E-05
Integrin mediated cell adhesion & migration	MELC, MRLC, MyHC, DOCK1, RASGRF1, Rac1, p190RhoGAP, LIMK2, Collagen IV, Zyxin, Actin cytoskeletal, MLCP (cat)	8.071E-05
Immune response by CD28 signaling	LAT, PIP5KI, Calcineurin A (catalytic), ZAP70, NF-AT, Rac1, PI3K cat class IA, CD86, Calmodulin, I-kB, Bcl-XL, p38 MAPK, Lck	9.403E-05

^1^pathways analysis performed using MetaCore software (St. Joseph, MI, USA)

**Table 3:** Over represented ontologies^1^ among genes dysregulated in the EOC.

** Ontology Network**	**Important Genes**	***p* value **
Cellular metabolic process	Calcineurin B1, NFIB, eIF1A, IL-11R, gp130, IL-27R, FOXK1, Tubulin alpha, PIP5KI, IL-22 SLC35C1, CYP4F12, Calnexin, FoxE3, δ-catenin, CYP3A5, CYP39A1, CDC14C, Cytochrome, c, GSTM1, NKIRAS1, IGF-1R, CDC20, MMP-7, CD45, HOXB5, GSTA1, CDK6, p16, CYP2F1	2.701E-16
Organic substance metabolic process	Calcineurin B1, NFIB, eIF1A, IL-11R, gp130, IL-27R, FOXK1, Tubulin alpha, PIP5KI, SLC35C1, CYP39A1, SLC16A11	3.291E-16
Primary metabolic process	CYP3A5, PKC, CYP39A1, CYP2A7, MMP-16, CYP19, SMAD3, CDC14C, Cytochrome c, VEGF-A, CDC25, COPS7, CDK6, CDK4, Calcineurin B1	1.509E-15
Cellular component organization	PIP5KI, c-Abl Stathmin, CDC25, GSPT2, MHC class II beta chain, HDAC4CDC20, WNT, IGFBP7/8	1.153E-13

^1^Gene ontology performed using MetaCore software (St. Joseph, MI, USA)

**Table 4:** The generation of biological networks with the shortest paths algorithm and relative saturation of networks with canonical pathways.

**Network Name**	**Processes**	**Size**	**Pathway**	***P* Value**	**z Score**	**g Score**
TRAF6, MyD88, I-kB, NIK(MAP3K14), MEK3(MAP2K3)	MyD88-dependent toll-like receptor signaling pathway (55.8%), toll-like receptor TLR6:TLR2 signaling pathway (53.8%), toll-like receptor TLR1:TLR2 signaling pathway (53.8%), toll-like receptor 2 signaling pathway (53.8%), toll-like receptor 4 signaling pathway (55.8%)	59	184	4.19e-09	9.61	239.61
TCF7L2 (TCF4), Dsh, Tcf(Lef), Axin, WNT	Wnt signaling pathway (58.0%), regulation of Wnt signaling pathway (58.0%), canonical Wnt signaling pathway (44.0%), regulation of canonical Wnt signaling pathway (52.0%), positive regulation of signal transduction (76.0%)	50	98	1.19e-14	14.09	136.59
ESR2, NFYA, CDC25A, SMAD3, TGF-β RII	negative regulation of transforming growth factor beta receptor signaling pathway (18.4%), N-acetylglucosamine metabolic process (12.2%), cellular response to endogenous stimulus (42.9%), regulation of transforming growth factor beta receptor signaling pathway (18.4%), response to endogenous stimulus (49.0%)	50	34	3.67e-22	19.26	61.76
