# Supplementary material for: Delineating Potential Transcriptomic Association with Organochlorine Pesticides in the Etiology of Epithelial Ovarian Cancer
Source: Open Biochem J. 2018 Feb 28;12:16–28. doi: 10.2174/1874091X01812010016 (PMC5848219; doi:10.2174/1874091X01812010016)
Supplement: Supplementary file 1 [file TOBIOCJ-12-16_SD1.pdf]

Send Orders for Reprints to reprints@benthamscience.ae

The Open Biochemistry Journal, 2018, *12*, i-ix

BENTHAM OPEN

CrossMark

The Open Biochemistry Journal

Supplementary Material

Content list available at: www.benthamopen.com/TOBIOCJ/

DOI: 10.2174/1874091X01812010016

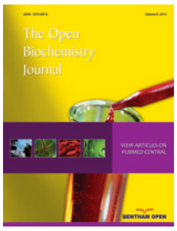

Delineating Potential Transcriptomic Association with Organochlorine Pesticides in the Etiology of Epithelial Ovarian Cancer

Harendra K. Shah[1], Muzaffer A. Bhat[3], Tusha Sharma[1], Basu D. Banerjee[1,*] and Kiran Guleria[2]

[1]*Environmental Biochemistry and Molecular Biology Laboratory, Department of Biochemistry, University College of Medical Sciences & G.T.B. Hospital (University of Delhi), Dilshad Garden, Delhi 110095, India*
[2]*Department of Obstetrics and Gynecology, University College of Medical Sciences & G.T.B. Hospital (University of Delhi), Dilshad Garden, Delhi 110095, India.*
[3]*Department of Physiology, All India Institute of Medical Sciences, New Delhi 110029, India*

Received: August 03, 2017 Revised: January 16, 2017 Accepted: January 30, 2017

superfine grinding of ovarian tissue (1gm)
↓
Acetone/Hexane for 30 minutes in shaker (5:2)
↓
Extract was filtered
↓
Same above procedure repeated once more time
↓
Extracted solvent was dried in rotary evaporator and dissolved in 5 ml of hexane
↓
Stored in freezer at -80^{0}C for 30 min to freeze the lipids
(Lipids precipitated as pale yellow)
↓
Immediately filtered with Whatman filter paper to remove frozen lipids
↓
Thus obtained filtered extract further cleaned up with activated florosil.
↓ Elution with 5 ml of acetone/hexane (1:9 v/v)
Finally concentrated to 1 ml
↓
Quantification of OCPs levels Gas Chromatograph (GC)

Fig. (1). Analytical procedure for the extraction and purification of OCPs from ovarian tissue samples.

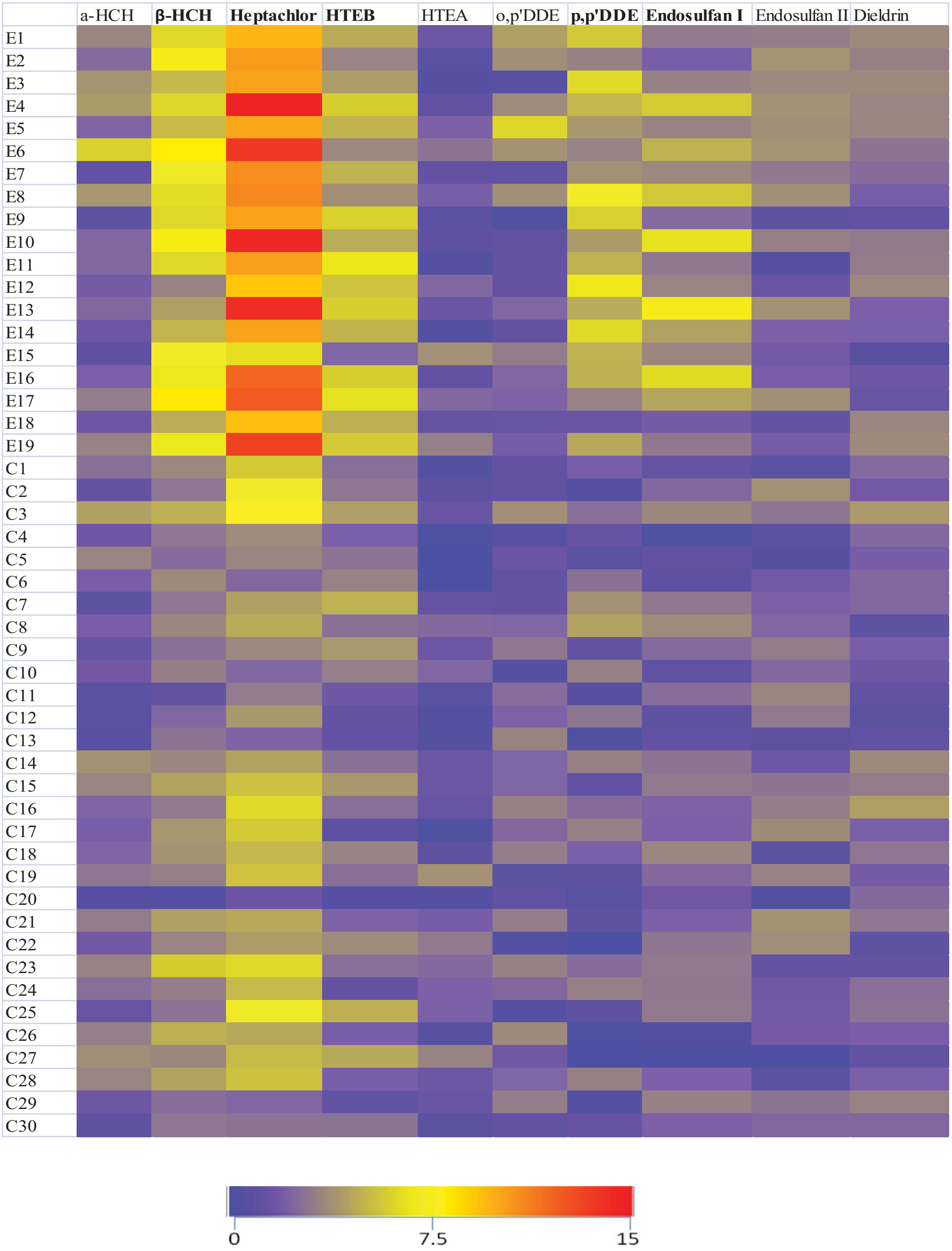

Fig. (2). Heat Map of distribution of different organochlorine pesticides (OCPs) in epithelial ovarian cancer and control ovary tissue samples.

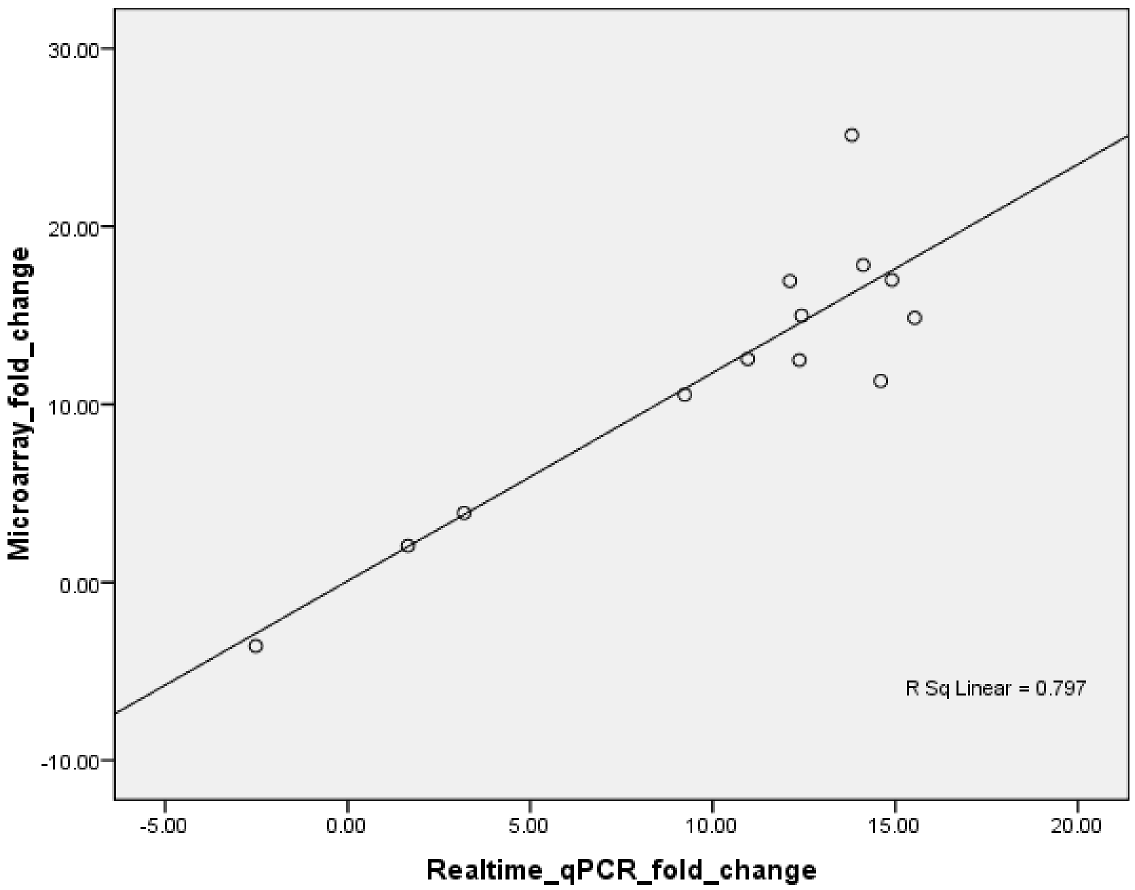

Fig. (3). Correlation between the Real time RT-PCR data and microarray fold change in epithelial ovarian cancer.

Supplementary Table 1. The comparison of various socio-demographic features in the two groups.

**Characteristics**	**Cases (n = 19)**	**Controls (n =30)**	**p-value**
**Age** (in years)	56.75 ± 7.18	53.06 ± 5.37	0.063
Mean±SD			
**BMI** (kg/m2)	27.12±2.33	24.07±4.25	0.006*
Mean±SD			
**Menopause**			
Yes	3 (10%)	6 (20%)	0.174
No	27 (90%)	24 (80%)	
**Dietary Habit**			
Vegetarian	20 (66.67%)	22 (56.67%)	0.637
Non-vegetarian	10 (33.33%)	8 (43.33%)	
**Water Resource**			
Govt. supply	21 (70%)	26 (86.67%)	0.067
Ground water	9 (30%)	4 (13.33%)	

Abbreviations: BMI, body mass index; SD, standard deviation;
*p-value<0.05 is considered as significant.

Supplementary Table 2. Summary of subject profiles enrolled in microarray experiment.

**Pt. ID**	**Age**	**cycle**[a]	**Onset of menarche**[b]	**Histopathology**		**Parity**	**Remark**	**Surgery**
				**Report**[c]	**Grade**[d]			
1	54	N	N	Clear cell carcinoma	3	P3L3	EOC	Uterovaginal prolapsed
2	65	Abn	N	Endometroid carcinoma	3	P2L2	EOC	Uterovaginal prolapsed

Suppl. Table 2 contd.....

Pt. ID	Age	cycle[a]	Onset of menarche[b]	Histopathology		Parity	Remark	Surgery
				Report[c]	Grade[d]			
3	47	N	N	Mucinous carcinoma	3	P2L2	EOC	Uterovaginal prolapsed
4	60	Abn	N	Endometroid	2	P3L3	EOC	Uterovaginal prolapsed
5	49	N	N	Papillary serous cystadenocarcinoma	3	P2L2	EOC	Uterovaginal prolapsed
6	45	Abn	N	Papillary serous cystadenocarcinoma	3	P3L3	EOC	Uterovaginal prolapsed
7	56	N	Late	Endometroid adenocarcioma	2	P3L3	EOC	Uterovaginal prolapsed
8	55	N	N	Clear cell carcinoma	3	P2L2	EOC	Uterovaginal prolapsed
9	40	N	N	Ovarian mass		P4L4	Control	Uterovaginal prolapsed
10	58	N	N	Ovarian cyst		P1L1	Control	Uteroveginal prolapsed
11	62	N	N	Ovarian cyst		P2L2	Control	Uteroveginal prolapsed
12	49	N	N	Ovarian mass		P1L1	Control	Uteroveginal prolapsed
13	41	N	N	Normal ovary		P1L1	Control	Uteroveginal prolapsed
14	42	N	N	Normal ovary		P2L2	Control	Uteroveginal prolapsed
15	48	N	N	Fibroma		P2L2	Control	Uteroveginal prolapsed

[a,b]based on the report obtained from the subject; Abn- Abnormal; Late- Late menopausal;
N- Normal
[c,d]based on histological reports.
Clinical severity stage 2 (mild), clinical severity stage 3 (moderate); 4, clinical severity stage 4 (severe)
P-total number of pregnancy; L- number of live offspring; EOC, epithelial ovary cancer

Supplementary Table 3. Some known function of Differential expressed genes in the high OCPs related to EOC.

Gene symbol	Gene name	Function
TGFβRII	transforming growth factor, beta receptor II	Performs many cellular functions, including the control of cell growth, cell proliferation.
NKIRAS1	NFKB inhibitor interacting Ras-like 1	Regulator of NF-κB activity
TXNRD2	Thioredoxinreductase 2	Regulate the cellular redox environment, defense against oxidative stress, play role in redox regulated cell signaling
CKLF	chemokine-like factor	Inflammatory response, ovarian cystadenoma
TNFRSF11A	tumor necrosis factor receptor superfamily, member 11a, NFKB activator	Induce activation of NF-κB activity & MAPK8/JAN.
PTPRC	Protein tyrosine phosphatase, receptor type, C	Regulate cellular process like mitosis, cell growth, differentiation, oncogenic transformation
IL37	Interleukin 37	Suppressor of innate inflammatory & immune response, involve in excessive inflammation
CCL23	chemokine (C-C motif) ligand 23	Immunoregulatory and inflammation
CCR2	Chemokine (C-C motif) receptor 2	Inflammatory response against tumors
G3BP2	GTPase activating protein (SH3 domain) binding protein 2	Includenodular malignant melanoma
HNMT	Histamine N-methyltransferase	Methylation and oxidative deamination
IGFBP7	Insulin-like growth factor binding protein 7	Cellular Senescence

Supplementary Table 4. The sets of sense (S) and antisense (AS) Primers used in the Real time RT-PCR.

	**Gene symbol, accession number and primer sequences[1]**	**Transcription activation of proto-oncogene, Error free DNA repair pathway, mediates transcriptional activation of target genes**
Gene Symbol	Accession number	Primer sequences[1]
	(mRNA RefSeq)	
TGF β RII	NM_001024847	S: AATATAACACCAGCAATC
		AS: AGATGATGATGACAGATA
NKIRAS1	NM_020345	S: GTAGTGTTAAGTAGGTTCATC
		AS: CTCAAGTATCTGGTCTATCA
TXNRD2	NM_000628	S: ACATCATCATTGCTACTG
		AS: ACTTGTGATTCCATATTCC
CKLF	NM_181641	S: TTGAAGTCACCGTTATCTTAT
		AS: CATACTGCTGTCACAAGT

Suppl. Table 4 contd.....

TNFRSF11A	NM_003839	S: TTGGCATTCTTCTTATTC
		AS: TTATTCTTCCTTGAACTTC
PTPRC	NR_052021	S: CATCATCACCTAGCAGTT
		AS: CTTATCAGACGAGGAACAA
IL37	NM_014439	S: GAACTTAAACCCGAAGAAAT
		AS: CTGGAACTGCTATGAGAT
CCL23	NM_005064	S: CCAGTGATAAGCAAGTTC
		AS: GACAAGTTCAATTCTTCCT
CCR2	NM_001123041	S: TCCTTGATGCTCATATTGTTC
		AS: TTCTCCACCTTCCATTCTT
G3BP2	NM_203505	S: AGGAGATATGGAACAGAATG
		AS: TTGGTATTGATGCGAAGT
HNMT	NM_006895	S: ATGTAATGTTGCCGTATAATCA
		AS: TCTCTATGCCAGTGTATTCC
IGFBP7	NM_001553	S: CAGTGGTTGATGCCTTAC
		AS: AAGTGTTAGTGGATTGGATT
UBE2V1	NM_001032288	S: TAGAAGATGACGAAGACA
		AS: ACCACTCCATTAGAACTAT
GAPDH	NM_002046	S: CCA AGG TCA TCC ATG ACA ACT TTG GT
		AS: TGT TGA AGT CAG AGG AGA CCA CCT G
β actin	NM_001101	S: TCA CAA GGA TTC CTA TGT GG
		AS: CTC ATT GTA GAA GGT GTG G

[1]Primers were designed using Beacon Designer Software (Premier Biosoft, Palo Alto, CA, USA) based on gene sequences corresponding to the genes, retrieved from NCBI data base.
AS- anti-sense ; S- sense.

Supplementary Table 5. The list of up and down-regulated gene set along with fold change among the differentially expressed gene sets between High (EOC) and Low (control) OCPs group.146 genes up-regulated and 13 genes Down-regulated identified by Welch T test at p <0.01 and FDR FC > 2.0.

**Up regulated genes in EOC**			
**Gene Name**	**Gene Symbol**	**Entrez**	**FC**
		**Gene ID**	
chemokine (C-C motif) receptor 2	CCR2	729230	25.13
DEAD (Asp-Glu-Ala-Asp) box polypeptide 10	DDX10	1662	24.88
MARCKS-like 1	MARCKSL1	65108	22.61
ribosomal protein L28	RPL28	6158	21.56
chromosome 20 open reading frame 26	C20orf26	26074	20.94
protocadherin gamma subfamily A, 2	PCDHGA2	56113	20.73
partner of NOB1 homolog (S. cerevisiae)	PNO1	56902	19.64
ankyrin repeat domain 53	ANKRD53	79998	19.05
KIAA1586	KIAA1586	57691	18.62
glucuronidase, beta pseudogene 1	GUSBP1	728411	18.6
zona pellucida glycoprotein 4	ZP4	57829	18.45
SLU7 splicing factor homolog (S. cerevisiae)	SLU7	10569	18.26
COP9 signalosome subunit 7A	COPS7A	50813	18.1
GTPase activating protein (SH3 domain) binding protein 2	G3BP2	9908	17.83
S100 calcium binding protein Z	S100Z	170591	17.79
dynein, axonemal, heavy chain 6	DNAH6	1768	17.37
potassium voltage-gated channel, subfamily H (eag-related), member 2	KCNH2	3757	17.3
mucolipin 1	MCOLN1	57192	17.2
dynein, axonemal, heavy chain 3	DNAH3	55567	17.02
protein tyrosine phosphatase, receptor type, C	PTPRC	5788	16.99
chemokine-like factor	CKLF	51192	16.93
olfactory receptor, family 4, subfamily S, member 1	OR4S1	256148	16.59
DNA cross-link repair 1A	DCLRE1A	9937	16.54

Suppl. Table 5 contd.....

**Up regulated genes in EOC**			
**Gene Name**	**Gene Symbol**	**Entrez**	**FC**
		**Gene ID**	
Kinocilin	KNCN	148930	16.47
FYVE, RhoGEF and PH domain containing 4	FGD4	121512	16.41
syntaxin 10	STX10	8677	16.23
Elastin	ELN	2006	16.22
solute carrier family 7, member 4	SLC7A4	6545	16.06
EPH receptor A7	EPHA7	2045	15.95
biogenesis of lysosomal organelles complex-1, subunit 2	BLOC1S2	282991	15.67
BCL6 corepressor pseudogene 1	BCORP1	286554	15.65
Stannin	SNN	8303	15.43
ubiquitin-conjugating enzyme E2 variant 1	UBE2V1	7335	14.99
interleukin 37	IL37	27178	14.86
ephrin-B3	EFNB3	1949	14.81
tetratricopeptide repeat domain 9C	TTC9C	283237	14.69
protein serine kinase H2	PSKH2	85481	14.61
integrin alpha FG-GAP repeat containing 2	ITFG2	55846	14.49
small integral membrane protein 9	SMIM9	1.00E+08	14.36
WAS/WASL interacting protein family, member 2	WIPF2	147179	14.18
ribosomal protein L10	RPL10	6134	14
Morf4 family associated protein 1	MRFAP1	93621	13.91
solute carrier family 22 (organic cation transporter), member 3	SLC22A3	6581	13.82
leucine rich repeat transmembrane neuronal 4	LRRTM4	80059	13.75
ATP synthase, H+ transporting, mitochondrial F1 complex, epsilon subunit	ATP5E	514	13.74
armadillo repeat gene deleted in velocardiofacial syndrome	ARVCF	421	13.69
olfactory receptor, family 4, subfamily C, member 15	OR4C15	81309	13.62
tudor domain containing 10	TDRD10	126668	13.31
chemokine-like factor	CKLF	51192	13.09
ST3 beta-galactoside alpha-2,3-sialyltransferase 3	ST3GAL3	6487	13.04
solute carrier family 16, member 11	SLC16A11	162515	13.02
ANKHD1-EIF4EBP3 readthrough	ANKHD1-EIF4EBP3	404734	13.01
CUB and Sushi multiple domains 3	CSMD3	114788	12.97
ribosomal protein L39	RPL39	6170	12.61
AT rich interactive domain 1A (SWI-like)	ARID1A	8289	12.59
thymus, brain and testes associated	TBATA	219793	12.56
chemokine (C-C motif) ligand 23	CCL23	6368	12.5
interleukin 10 receptor, beta	IL10RB	3588	12.51
histamine N-methyltransferase	HNMT	3176	12.49
mannosyl (alpha-1,3-)-glycoprotein beta-1,4-N-acetylglucosaminyltransferase, isozyme C (putative)	MGAT4C	25834	12.47
PX domain containing serine/threonine kinase	PXK	54899	12.45
paired box 7	PAX7	5081	12.43
transmembrane protein 167A	TMEM167A	153339	12.43
psoriasis susceptibility 1 candidate 1	PSORS1C1	170679	12.37
zinc finger protein 596	ZNF596	169270	12.36
solute carrier family 25, member 39	SLC25A39	51629	12.23
uncoupling protein 2 (mitochondrial, proton carrier)	UCP2	7351	12.1
nuclear factor, erythroid 2-like 1	NFE2L1	4779	12.07
solute carrier family 8 (sodium/calcium exchanger), member 3	SLC8A3	6547	12.03
ubiquitin specific peptidase 20	USP20	10868	11.87
2,3-bisphosphoglycerate mutase	BPGM	669	11.85
phosphopantothenoylcysteine decarboxylase	PPCDC	60490	11.72
nth endonuclease III-like 1 (E. coli)	NTHL1	4913	11.66
short chain dehydrogenase/reductase family 42E, member 1	SDR42E1	93517	11.64
tubulin, alpha-like 3	TUBAL3	79861	11.6

Suppl. Table 5 contd.....

**Up regulated genes in EOC**			
**Gene Name**	**Gene Symbol**	**Entrez**	**FC**
		**Gene ID**	
G protein-coupled receptor associated sorting protein 2	GPRASP2	114928	11.42
NFKB inhibitor interacting Ras-like 1	NKIRAS1	28512	11.31
N-acetylglucosamine-1-phosphate transferase, alpha and beta subunits	GNPTAB	79158	11.19
family with sequence similarity 222, member A	FAM222A	84915	11.15
ubiquitin specific peptidase 2	USP2	9099	10.98
RNA binding protein S1, serine-rich domain	RNPS1	10921	10.95
sema domain, immunoglobulin domain (Ig), short basic domain, secreted, (semaphorin) 3G	SEMA3G	56920	10.86
MED14 opposite strand	MED14OS	1.01E+08	10.74
TIA1 cytotoxic granule-associated RNA binding protein	TIA1	7072	10.62
thioredoxin reductase 2	TXNRD2	10587	10.54
ZFP57 zinc finger protein	ZFP57	346171	10.38
KAT8 regulatory NSL complex subunit 2	KANSL2	54934	10.3
secretoglobin, family 1D, member 1	SCGB1D1	10648	10.25
methyltransferase like 2A	METTL2A	339175	9.96
stathmin 1	STMN1	3925	9.7
synovial sarcoma, X breakpoint 4B	SSX4B	548313	9.62
GDP dissociation inhibitor 2	GDI2	2665	9.53
mediator complex subunit 11	MED11	400569	9.42
GRIP and coiled-coil domain containing 2	GCC2	9648	9.38
Kv channel interacting protein 1	KCNIP1	30820	9.29
DnaJ (Hsp40) homolog, subfamily C, member 27	DNAJC27	51277	9.28
solute carrier family 2 (facilitated glucose transporter), member 8	SLC2A8	29988	9.15
synaptonemal complex protein 1	SYCP1	6847	9.08
chromosome 11 open reading frame 57	C11orf57	55216	9.08
gap junction protein, gamma 3, 30.2kDa	GJC3	349149	8.71
vestigial-like family member 2	VGLL2	245806	8.17
polymerase (RNA) III (DNA directed) polypeptide H (22.9kD)	POLR3H	171568	8.03
family with sequence similarity 136, member A	FAM136A	84908	7.89
UTP20, small subunit (SSU) processome component, homolog (yeast)	UTP20	27340	7.69
TBC1 domain containing kinase	TBCK	93627	7.48
polymerase (RNA) mitochondrial (DNA directed)	POLRMT	5442	7.48
WAS protein family homolog 1	WASH1	1.00E+08	7.35
homeobox A3	HOXA3	3200	5.76
oxysterol binding protein-like 10	OSBPL10	114884	5
Syntaphilin	SNPH	9751	4.92
family with sequence similarity 27, member C	FAM27C	1.00E+08	4.84
androgen-dependent TFPI-regulating protein	ADTRP	84830	4.47
phospholipase A2, group XVI	PLA2G16	11145	4.29
phosphatidylserine decarboxylase	PISD	23761	4.22
potassium voltage-gated channel, subfamily H (eag-related), member 8	KCNH8	131096	4.05
equatorin, sperm acrosome associated	EQTN	54586	4.05
sorting nexin 15	SNX15	29907	4.04
tripartite motif containing 66	TRIM66	9866	3.96
tumor necrosis factor receptor superfamily, member 11a, NFKB activator	TNFRSF11A	8792	3.9
Smith-Magenis syndrome chromosome region, candidate 8	SMCR8	140775	3.79
THO complex 2	THOC2	57187	3.7
ORMDL sphingolipid biosynthesis regulator 2	ORMDL2	29095	3.73
purinergic receptor P2X, ligand-gated ion channel, 5	P2RX5	5026	3.72
zinc finger protein 283	ZNF283	284349	3.72
2-oxoglutarate and iron-dependent oxygenase domain containing 2	OGFOD2	79676	3.63
phosphatidylinositol-4-phosphate 5-kinase, type I, beta	PIP5K1B	8395	3.57
zinc finger protein 300	ZNF300	91975	3.49

Suppl. Table 5 contd.....

Up regulated genes in EOC			
**Gene Name**	**Gene Symbol**	**Entrez**	**FC**
		**Gene ID**	
potassium voltage-gated channel, subfamily H (eag-related), member 2	KCNH2	3757	3.38
zinc and ring finger 3	ZNRF3	84133	3.25
transcription factor 7-like 2 (T-cell specific, HMG-box)	TCF7L2	6934	3.23
exportin, tRNA	XPOT	11260	3.18
G protein-coupled receptor 27	GPR27	2850	3.17
chromosome 8 open reading frame 44	C8orf44	56260	3.087
mab-21-like 3 (C. elegans)	MAB21L3	126868	3.02
transmembrane protein 128	TMEM128	85013	2.99
SIVA1, apoptosis-inducing factor	SIVA1	10572	2.88
protein kinase (cAMP-dependent, catalytic) inhibitor gamma	PKIG	11142	2.8
ankyrin repeat domain 13A	ANKRD13A	88455	2.61
transforming growth factor, beta receptor II (70/80kDa)	TGFBR2	7048	2.39
thioesterase superfamily member 4	THEM4	117145	2.27
sperm associated antigen 9	SPAG9	9043	2.27
proliferating cell nuclear antigen	PCNA	5111	2.26
MYC binding protein 2, E3 ubiquitin protein ligase	MYCBP2	23077	2.21
Down regulated genes in EOC			
interleukin 13 receptor, alpha 1	IL13RA1	3597	-2.05
putative protein FAM90A7	FAM90A7P	441317	-2.12
solute carrier family 44 (choline transporter), member 2	SLC44A2	57153	-2.22
elongator acetyltransferase complex subunit 6	ELP6	54859	-2.2
isoprenoid synthase domain containing	ISPD	729920	-2.3
chromosome 2 open reading frame 69	C2orf69	205327	-2.54
component of oligomeric golgi complex 8	COG8	84342	-2.55
alpha tubulin acetyltransferase 1	ATAT1	79969	-2.58
spermatid maturation 1	SPEM1	374768	-2.58
piezo-type mechanosensitive ion channel component 2	PIEZO2	63895	-2.92
dual specificity phosphatase 2	DUSP2	1844	-3.53
insulin-like growth factor binding protein 7	IGFBP7	3490	-3.58
transmembrane protein 150C	TMEM150C	441027	-6.35

Supplementary Table 6. Correlation analysis between the differential gene expression and higher level of OCPs among healthy and EOC patients.

Genes	αHCH	βHCH	Heptachlor	HTEB	HTEA	opDDE	ppDDE	Endo1	Endo2	Dieldrin
**TgfβRII (p)**	0.249	-0.054	-0.02	-0.11	0.168	-0.148	-0.317	-0.143	-0.149	0.244
**(S)**	0.132	0.748	0.929	0.51	0.313	0.377	0.052	0.393	0.372	0.14
**Nkiras1 (p)**	-0.148	-.706**	-.807**	-.655**	-0.188	-0.138	-.659**	-.601**	-0.227	-0.233
**(S)**	0.374	0	0	0	0.257	0.409	0	0	0.171	0.159
**Txnrd2 (p)**	-0.064	-.649**	-.677**	-.576**	-0.179	-0.085	-.702**	-.461**	-0.171	-0.063
**(S)**	0.704	0	0	0	0.283	0.613	0	0.004	0.305	0.707
**Cklf (p)**	-0.119	-.741**	-.793**	-.736**	-0.249	-0.223	-.684**	-.592**	-0.244	-0.196
**(S)**	0.477	0	0	0	0.132	0.178	0	0	0.14	0.237
**Tnfrsf11a(p**	0.207	-.382*	-.402*	-.468**	-0.053	-0.102	-.551**	-0.288	0.09	0.128
**(S)**	0.213	0.018	0.012	0.003	0.752	0.54	0	0.079	0.592	0.443
**Ptprc (p)**	-0.185	-.745**	-.754**	-.563**	-0.13	-0.213	-.675**	-.581**	-0.128	-0.059
**(S)**	0.267	0	0	0	0.436	0.2	0	0	0.444	0.725
**Il37 (p)**	-0.15	-.750**	-.780**	-.649**	-0.221	-0.015	-.590**	-.517**	-0.143	-0.104
**(S)**	0.368	0	0	0	0.182	0.928	0	0.001	0.39	0.536
**Ccl23 (p)**	-0.289	-.691**	-.745**	-.567**	-0.307	-0.159	-.586**	-.431**	-0.188	-0.225
**(S)**	0.079	0	0	0	0.061	0.339	0	0.007	0.26	0.174
**Ccr2 (p)**	-0.161	-.685**	-.768**	-.656**	-.359*	-0.113	-.613**	-.611**	-0.156	-0.156

Suppl. Table 6 contd.....

Genes	αHCH	βHCH	Heptachlor	HTEB	HTEA	opDDE	ppDDE	Endo1	Endo2	Dieldrin
**(S)**	0.334	0	0	0	0.027	0.501	0	0	0.35	0.349
**G3bp2 (p)**	-0.212	-.624**	-.634**	-.463**	-0.116	-0.188	-.541**	-.350*	-0.191	-0.107
**(S)**	0.201	0	0	0.003	0.487	0.257	0	0.031	0.25	0.525
**Hnmt (p)**	-0.07	-.653**	-.680**	-.626**	-0.101	0.011	-.585**	-.456**	-0.1	0.03
**(S)**	0.677	0	0	0	0.546	0.946	0	0.004	0.55	0.856
**IGFBP7 (p)**	0.263	.628**	.561**	0.318	0.29	-0.034	.376*	.484**	-0.048	-0.098
**(S)**	0.11	0	0	0.052	0.077	0.842	0.02	0.002	0.774	0.56
**UBE2V1 (p)**	-0.122	-.690**	-.739**	-.604**	-0.225	-0.173	-.632**	-.490**	-.336*	-0.157
**(S)**	0.467	0	0	0	0.175	0.3	0	0.002	0.039	0.347

(p) Pearson Correlation
(s) Sig. (2-tailed)
Endo1: endosulfan 1; Endo2: endosulfan 2
**. Correlation is significant at the 0.01 level (2-tailed).
*. Correlation is significant at the 0.05 level (2-tailed).

© 2018 Shah *et al*.

This is an open access article distributed under the terms of the Creative Commons Attribution 4.0 International Public License (CC-BY 4.0), a copy of which is available at: https://creativecommons.org/licenses/by/4.0/legalcode. This license permits unrestricted use, distribution, and reproduction in any medium, provided the original author and source are credited.
